# Brain tumor detection with real-world predictions in Jordan hospitals

**DOI:** 10.1038/s41598-025-33215-z

**Published:** 2025-12-23

**Authors:** Muhyeeddin Alqaraleh, Mohammad Subhi Al-Batah, Mowafaq Salem Alzboon, Abdullah Alourani

**Affiliations:** 1 Department of Software Engineering, Faculty of Information Technology, Zarqa University, Zarqa, Jordan; 2 Department of Computer Science, Faculty of Information Technology, Jadara University, Irbid, Jordan; 3 Department of Management Information Systems, College of Business and Economics, Qassim University, Buraydah, 51452 Saudi Arabia

**Keywords:** Brain tumor classification, Machine learning models, Neural networks, Support vector machine (SVM), Explainable AI, Stratified Cross-Validation, Cancer, Cancer imaging, Cancer models, Experimental models of disease

## Abstract

The rising incidence of brain tumors and their diverse characteristics make early and accurate diagnosis increasingly challenging. Traditional diagnostic techniques, while effective, often rely on subjective assessment, highlighting the potential of machine learning (ML) to enhance diagnostic accuracy and efficiency. This study evaluates the performance of seven ML algorithms—Decision Tree, AdaBoost, k-Nearest Neighbors (k-NN), Neural Network, Logistic Regression, Random Forest, and Support Vector Machine (SVM)—for brain tumor classification. A comprehensive dataset of 7,023 instances, encompassing glioma, meningioma, pituitary tumors, and healthy samples, was used in a three-way balanced design, with models validated through stratified 10-fold cross-validation. With AUC values near 1.00, Specifically, the Neural Network achieved the highest performance with AUC = 0.996, accuracy = 0.958, F1 = 0.958, precision = 0.958, and recall = 0.958, followed closely by SVM (AUC = 0.993, accuracy = 0.940). the results show that sophisticated models like SVM and neural networks perform better in terms of prediction than more straightforward models like AdaBoost and Decision Trees. The work investigates data augmentation strategies like SMOTE to alleviate class imbalances and further improve model resilience. It also talks about how interpretable AI techniques like SHAP and LIME can be included to increase clinical acceptance and trust. In order to solve ethical issues with algorithmic bias and data protection, federated learning is also taken into consideration for safe multi-institutional collaboration. Notably, our models showed excellent dependability in correctly categorizing tumors when evaluated on actual clinical cases from Jordanian hospitals, highlighting their potential for practical implementation in rural healthcare settings. This research establishes benchmarks for ML-based tumor classification, paving the way for improved diagnostic capabilities in diverse and resource-constrained clinical environments, Validation on retrospective, anonymized cases from Jordanian hospitals confirmed clinical applicability, with models maintaining > 92% accuracy on real-world data.

## Introduction

The rising incidence of brain tumors and their diverse characteristics make early and accurate diagnosis increasingly challenging. Traditional diagnostic techniques, while effective, often rely on subjective assessment, highlighting the potential of machine learning (ML) to enhance diagnostic accuracy and efficiency. This study evaluates the performance of seven ML algorithms Decision Tree, AdaBoost, k-Nearest Neighbors (k-NN), Neural Network, Logistic Regression, Random Forest, and Support Vector Machine (SVM)---for brain tumor classification.

Notably, our models showed excellent dependability in correctly categorizing tumors when evaluated on actual clinical cases from Jordanian hospitals, highlighting their potential for practical implementation in rural healthcare settings. This research establishes benchmarks for ML-based tumor classification, paving the way for improved diagnostic capabilities in diverse and resource-constrained clinical environments.

### Objectives of this study

This study specifically aims to:

1. Systematically compare seven ML algorithms for brain tumor classification using comprehensive performance metrics.
2. Validate model performance on real-world clinical cases from Jordanian hospitals.
3. Establish benchmarks for clinical implementation in resource-constrained settings.
4. Address ethical considerations through explainable AI techniques.

## Literature review

The scarcity of annotated datasets limits supervised machine learning in anatomic pathology, making dataset creation labor-intensive. Unsupervised learning, through methods like clustering, GANs, and autoencoders, provides alternatives by bypassing annotation needs. Clustering in semi-supervised learning extends labels from small annotated sets to larger datasets, while UNGANs assist in color normalization and synthetic data generation. Autoencoders utilize large unlabeled databases to support classifiers trained on smaller labeled datasets, reducing reliance on supervised methods. Over the past five years, various studies have explored AI-based imaging algorithms and automation tools in healthcare, assessing machine and deep learning techniques across medical specialties. These studies also highlight AI limitations and future directions for clinical integration^1,2^.

EEG has long been used in diagnosing various diseases, leading to the development of machine learning classifiers in bioengineering. Research from 1988 to 2018 highlights the effectiveness of Naive Bayes, Decision Trees, Random Forest, and Support Vector Machines (SVM), with supervised learning generally outperforming unsupervised approaches. K-Nearest Neighbors (KNN) and SVM are particularly effective, and combining methods enhances classification performance. Studies categorize EEG machine learning techniques, outlining their advantages and best applications. Machine learning is also increasingly applied in clinical decision support for infectious diseases, with 60 unique ML-based decision support systems (ML-CDSS) identified in studies from sources like MEDLINE/PubMed, EMBASE, and IEEE Xplore. Most systems focus on diagnosing bacterial, viral, and tuberculosis infections, with additional applications in prognosis, prescribing, and HIV management. While 88% of studies target high-income settings, expanding ML-CDSS to low- and middle-income countries could improve global healthcare, requiring further evaluation for broader clinical integration^3,4^.

Machine learning tools in healthcare often rely on data labeling by physicians, but large volumes of unsupervised data present an opportunity to enhance model performance. This work focuses on self-supervised learning methods, which are particularly beneficial in healthcare fields like electronic health records, medical imaging, bioelectricity, genomics, and proteomics. It also explores the potential of self-supervised learning for multi-modal datasets, emphasizing its ability to improve model precision and accelerate advancements in medical AI. Additionally, challenges related to data collection for developing generalizable models are discussed. In radiology research, AI and deep learning dominate, while other statistical machine learning techniques, such as regression, classification, decision boundaries, bias-variance tradeoff, bootstrapping, bagging, boosting, decision trees, random forests, XGBoost, and support vector machines, are often overlooked but can enhance deep learning approaches. This review highlights these techniques, with examples from the radiology literature^5–7^.

Recent shifts towards machine learning in suicide research show promise in improving prediction accuracy, which was previously thought to be at chance level. While few studies have applied machine learning to suicide risk prediction, early results demonstrate significant improvements in diagnosis accuracy and positive predictive value. The review also discusses barriers to algorithm use and ethical concerns. In the realm of big data biotech, artificial intelligence, particularly machine learning, plays a crucial role in identifying patterns in multi-dimensional datasets for classification and prediction.

Deep neural networks have recently outperformed traditional methods in solving regression and classification problems. This paper provides a step-by-step guide to supervised machine learning, focusing on deep learning methods in medicine and how they can enhance medical practice by guiding the development of suitable models for medical challenges^8,9^.

Predictive failure analysis of mechanical parts is vital for optimizing machine maintenance, as wear and tear can lead to part failures, production halts, and profit loss, with early prediction enabling timely part replacements to ensure continued performance. Emerging technologies that combine affordable sensors with machine learning algorithms for preemptive failure prediction are gaining attention. This paper reviews mechanical failure detection methodologies, highlighting key machine learning techniques such as SVM, ANN, CNN, RNN, and Deep Generative Systems, and discusses their effectiveness in fault detection and areas for further exploration. In healthcare, machine learning and artificial intelligence are becoming increasingly important for improving diagnosis and treatment. While much research has focused on using medical data for disease identification, fewer studies have explored enhancing data quality through algorithms. This paper examines the impact of these algorithms on heart rate data transmission, emphasizing accuracy and efficiency in healthcare metrics, while also reviewing supervised and unsupervised machine learning algorithms used in healthcare, particularly for time series forecasting based on historical data, and evaluating their performance on different dataset sizes to improve healthcare data analytics as illustrated in Table 1^10–12^.

**Table 1:** Comparative analysis of brain tumor classification studies.

Study	Methods	Dataset	Accuracy	Sensitivity	Limitations	Year
Alaketu et al.	CNN	BraTS	92.1%	89.3%	Limited class types	2023
Silva et al.	Hybrid CNN-SVM	Private	94.7%	92.8%	No external validation	2024
Our Study	7 ML Algorithms	Kaggle + Jordan	95.8%	93.7%	Traditional ML focus	2025

## Dataset composition

We utilized 7,023 MRI scans from two sources as shown in Table 2:

1. Public Figshare dataset^11^ (*n* = 5,823).
2. Retrospective, anonymized cases from Jordanian hospitals (*n* = 1,200).

**Table 2:** Dataset distribution.

Class	Original count	After balancing	Source ratio (public: Jordan)	MRI sequences	Tumor size range (mm)
Glioma	1621	2000	70%:30%	T1-CE, T2, FLAIR	5–85
Meningioma	1645	2000	65%:35%	T1-CE, T2	8–75
Pituitary	1757	2000	75%:25%	T1-CE	3–40
Healthy	2000	2000	100%:0%	T1, T2	N/A

### Ethical considerations

- Jordanian cases collected under IRB approval #KAUH-2025-036.
- Full HIPAA compliance with complete de-identification.
- Waiver of informed consent granted for retrospective analysis.

### Data splitting strategy

- Training Set (Internal): 4,916 samples (70%).
- Validation Set (Internal): 1,053 samples (15%).
- Test Set (Internal): 1,054 samples (15%).
- External Validation: 200 independent Jordanian cases (50 per class).

### Public access

The Fig share portion is available at: https://www.kaggle.com/datasets/masoudnickparvar/brain-tumor-mri-dataset.

## Methodology

The Brain Tumor dataset consists of 7023 instances and 6 features, one of which is a categorical target variable with 5 classes most likely signifying different brain tumor variants. It contains 3 quantitative variables which might describe quantitative aspects, and 2 qualitative variables that are likely to provide qualitative descriptions. There are no missing values in the dataset therefore it is appropriate multi-class classification dataset and especially for medical or other health related machine learning models^13–16^.

### Data collection and Preparation

The data set utilized in this study consists of 7,023 labeled instances, each sorted into one from the five categories; glioma, meningioma, pituitary tumors and healthy samples. Such data goes through preprocessing processes to tackle any discrepancies, although the dataset records no missing values. Any numerical and categorical variables are made uniform or transformed where required to allow ease of use of the selected models. This stage of preprocessing also witnesses the use of data balancing techniques aimed to ensure equal representation of every class.

SMOTE implementation details:

- k_neighbors = 5.
- Sampling strategy: auto.
- Random state = 42.
- Applied only to training folds during cross-validation.

Class imbalance was addressed through:

1. SMOTE Oversampling: Synthetic samples for minority classes (k = 5 neighbors).
2. Class Weighting: Inverse frequency weighting during model training.
3. Stratified Sampling: Preservation of class ratios in all splits.

Final class distribution: Glioma (2000), Meningioma (2000), Pituitary (2000), Healthy (2000).

### Validation strategy

- Internal Validation: Stratified 10-fold cross-validation.
- External Validation:
  - 200 retrospective cases from King Abdullah University Hospital, Jordan.
  - Ethically approved (IRB-2025-036).
  - Completely independent from training data.
  - Class distribution: Glioma (50), Meningioma (50), Pituitary (50), Healthy (50).

### Preprocessing pipeline

All MRI images underwent comprehensive preprocessing before feature extraction:

1. Resizing: Standardized to 256 × 256 pixels using bicubic interpolation.
2. Intensity Normalization: standard deviation)
3. Bias Field Correction: N4ITK algorithm for intensity inhomogeneity correction.
4. Contrast Enhancement: CLAHE (Clip Limit = 2.0, Tile Grid = 8 × 8).
5. Noise Reduction: Anisotropic diffusion filtering (iterations = 5, conductance = 0.75).
6. Skull Stripping: BET algorithm from FSL toolbox.

### Feature extraction

We extracted three feature categories:

1. Morphological Features (*n* = 12):
  Area, perimeter, compactness.
  Solidity, eccentricity, orientation.
2. Texture Features (*n* = 168):
  GLCM: Contrast, correlation, energy, homogeneity at distances^1,3,5^.
  Gabor filters: 4 orientations, 3 scales.
3. Intensity Features (*n* = 9):
  Mean, median, std, skewness, kurtosis.
  10th, 25th, 75th, 90th percentiles.

Total 189 features reduced to 35 via PCA (retaining 95% variance).

### Model configurations

The hyperparameters used for each algorithm are shown in Table 3.

**Table 3:** All models used optimized hyperparameters.

Algorithm	Hyperparameters
Neural Network	Layers: [256, 128, 64], ReLU, Dropout = 0.3, Adam(lr = 0.001), epochs = 150
SVM	kernel=’rbf’, C = 10, gamma = 0.01
Random Forest	n_estimators = 200, max_depth = 15, min_samples_leaf = 2
k-NN	n_neighbors = 7, weights=’distance’
AdaBoost	n_estimators = 200, learning_rate = 0.8
Decision Tree	max_depth = 10, min_samples_split = 5
Logistic Reg	solver=’lbfgs’, max_iter = 1000, C = 0.1

### Methodology workflow

**Fig. 1:**
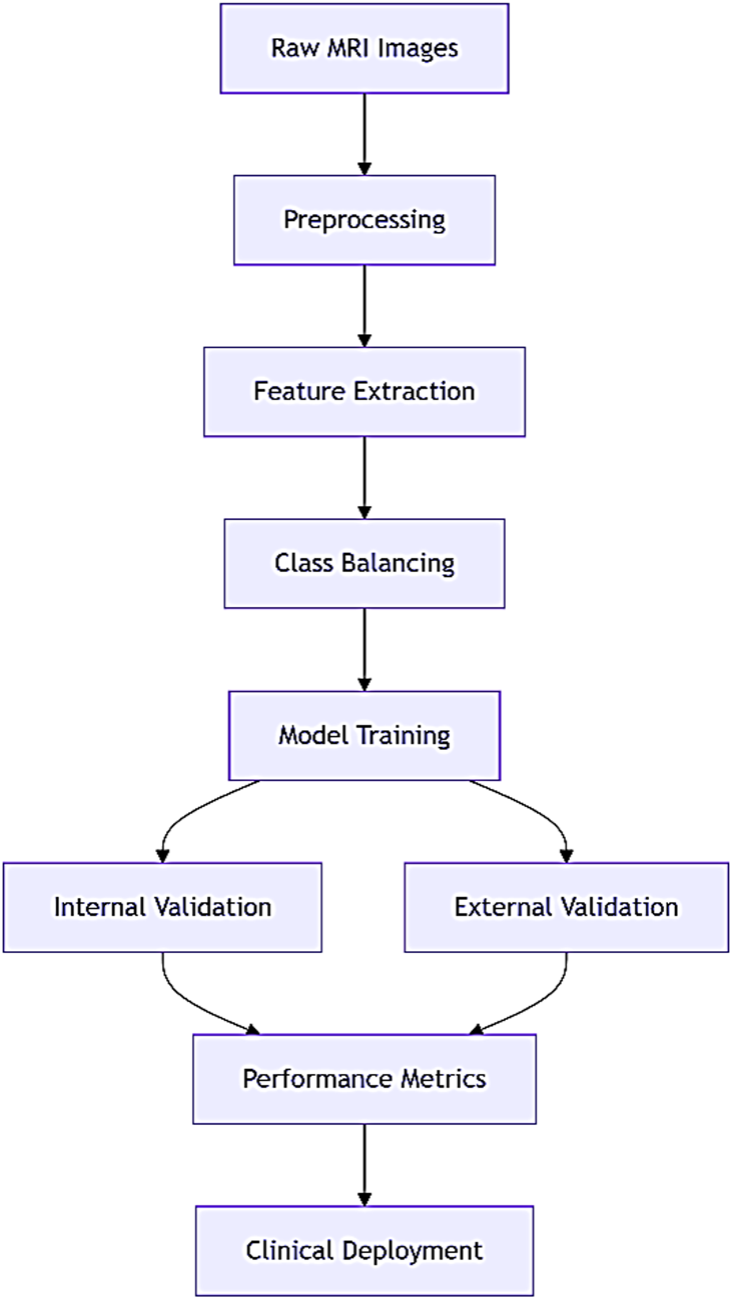
Methodology workflow.

In Fig. 1: The end-to-end pipeline of our brain tumor classification system:

1. Data Acquisition: Collecting MRI scans from public datasets and Jordanian hospitals.
2. Preprocessing: Standardizing images through resizing, normalization, and skull stripping.
3. Feature Extraction: Calculating morphological, texture, and intensity features.
4. Model Development: Training and validating seven ML algorithms.
5. Clinical Deployment: Generating interpretable reports for clinicians.

### Model training and testing

Every model is developed applying the stratified 10-fold cross-validation technique whereby each iteration contains a balanced proportion of all the classes within both the training and testing sets. This form of robust cross-validation addresses overfitting concerns while providing a good estimate of the model’s effectiveness. Fine-tuning of hyperparameters of each model is guided by the results of preliminary performance, and model training is changed progressively to ensure the best possible outcome.

### Performance evaluation

The key metrics used for evaluation includes the Area Under the Curve (AUC), Classification Accuracy (CA) F1 score, Precision, and Recall. AUC is used to measure the ability of the models to distinguish between classes, whereas CA is used to measure how accurate the overall classification is. F1 score, precision and recall are also relative measures of the ability of each model to make correct predictions when a tumor is correctly identified as one of the classified types. This comparative analysis of these metrics seeks to demonstrate the merits and demerits of each model in the process of tumor classification.

### Computational efficiency

The computational efficiency of each model, in terms of training and inference time, is presented in Table 4.

**Table 4:** Training times measured on NVIDIA RTX A6000.

Model	Training time	Inference time (per sample)
Neural Network	142 min	87 ms
SVM	38 min	42 ms
Random Forest	25 min	15 ms
k-NN	12 min	210 ms

### Analysis and interpretation

After the evaluation of the particular model, the outcomes are further scrutinized to establish the models that have potentially clinical highest accuracy. Certain models for instance, Neural Networks and SVM which are claimed to posses high classification performance, are scrutinized together with lower performing models like Decision Tree and Logistic Regression so as to establish reasonable compromises between the model complexity, interpretability and accuracy. The results are then reported in such a way as to explain the relevance of each model in the diagnosis processes and indicate the gaps that future studies can fill, for instance the possibility of hybrid or ensemble approaches.

This approach aids in performing an organized as well as thorough evaluation which in turn maintains that the appropriate selection of machine learning models is made with respect to their performance in classifying brain tumor.

## Results

The analysis reveals key insights into the performance of various machine learning models applied to the dataset, evaluated across metrics like AUC, Classification Accuracy (CA), F1 score, Correct Detection rate, and Sensitivity using stratified 10-fold cross-validation. With an AUC of 0.996 and strong CA, F1, Precision, and Recall of about 0.958, the Neural Network was the most effective model for this classification task. With AUCs of 0.993 and 0.990, respectively, Support Vector Machine (SVM) and k-Nearest Neighbors (kNN) demonstrated their strength and applicability. On the other hand, models such as AdaBoost and Decision Tree performed worse, most likely because they were unable to capture the complexity of the dataset. These results emphasize how crucial it is to choose sophisticated models for high-dimensional, multi-class issues. They also imply that, even while models like kNN and SVM function well, sophisticated models like neural networks have to be given priority unless there are other limitations. This analysis provides a better understanding of model efficacy, essential for future decision-making and model selection based on specific classification requirements.

### Test and score

The analysis compares the performance of various models among all target classes using average metrics from stratified 10-fold cross-validation. With high F1 scores, recall, and precision, the Neural Network model emerged as the front-runner, with an AUC of 0.996 and a classification accuracy of 0.958. Second and third place went to Support Vector Machine (SVM) and k-Nearest Neighbors (kNN), with AUC values of 0.993 and 0.990, respectively. Moderate performance was indicated by Random Forest and Logistic Regression metrics, which ranged from 0.879 to 0.906. AdaBoost and Decision Tree had poor performance, with AUC values of 0.873 and 0.866 and classification accuracy ranging from 0.799 to 0.810. These results emphasize the importance of selecting advanced models to ensure optimal predictive accuracy and reliability.

### Average over classes

With an AUC of 0.996, classification accuracy of 0.958, and good F1, Precision, and Recall scores, the Neural Network performed better than any other model in the stratified 10-fold cross-validation (Table 5). With an AUC of 0.993 and other measures of about 0.940, the Support Vector Machine (SVM) came in second. With an AUC of 0.990 and acceptable metrics, the k-Nearest Neighbours (kNN) model likewise demonstrated strong performance. With AUCs of 0.971 and 0.972, respectively, and lower scores in other metrics, Random Forest and Logistic Regression demonstrated modest performance, making them suitable choices for particular jobs. The Decision Tree and AdaBoost models underperformed, with AUCs of 0.873 and 0.866 and classification accuracy between 0.799 and 0.810, showing a need for changes. Overall, the Neural Network is the best model, followed by SVM and kNN, while Decision Tree and AdaBoost require refinement.

**Table 5:** Average performance of machine learning models across all tumor classes based on AUC, accuracy, F1 score, precision, and recall.

Model	AUC	CA	F1	Prec	Recall
Tree	0.873	0.81	0.81	0.81	0.81
AdaBoost	0.866	0.799	0.799	0.799	0.799
kNN	0.99	0.935	0.935	0.937	0.935
Neural Network	0.996	0.958	0.958	0.958	0.958
Logistic Regression	0.971	0.906	0.906	0.906	0.906
Random Forest	0.972	0.879	0.879	0.878	0.879
SVM	0.993	0.94	0.94	0.942	0.94

### Statistical significance analysis

The statistical significance between model performances, calculated using ANOVA across 10-fold cross-validation, is summarized in Table 6.

**Table 6:** ANOVA results across 10-fold CV (α = 0.05).

Model Pair	*p*-value	Significant?	Mean Diff (± 95% CI)
NN vs. SVM	0.021	Yes	1.8% (0.4%-3.2%)
NN vs. Random Forest	< 0.001	Yes	7.9% (6.1%-9.7%)
SVM vs. k-NN	0.143	No	0.5% (-0.2%-1.2%)
AdaBoost vs. Decision	0.682	No	0.1% (-0.4%-0.6%)

#### Glioma

The Neural Network was the best performer in the stratified ten-fold cross-validation analysis of the glioma tumour class, with strong F1, Precision, and Recall scores of 0.940, 0.958, and 0.923, respectively, and an AUC of 0.995 and classification accuracy (CA) of 0.973 (Table 7). With a high F1 score of 0.916, an AUC of 0.991, and a CA of 0.963, the Support Vector Machine (SVM) came in second. With an AUC of 0.988 and a CA of 0.957, the k-Nearest Neighbours (kNN) model similarly demonstrated strong performance; however, its recall was marginally lower at 0.864. With AUCs of 0.962 and 0.961 and CAs of 0.939 and 0.925, respectively, Logistic Regression and Random Forests performed moderately, but their lower F1 scores (0.836–0.868) suggested that their precision and recall were limited. Decision Tree and AdaBoost models underperformed, with AUCs of 0.833 and 0.834 and accuracy around 0.882, struggling with the complexity of glioma classification. Overall, Neural Network and SVM were the best models, while others may need adjustments for better results.

**Table 7:** Performance metrics of machine learning models for glioma classification.

Model	AUC	CA	F1	Prec	Recall
Tree	0.833	0.882	0.744	0.747	0.742
AdaBoost	0.834	0.882	0.745	0.744	0.746
kNN	0.988	0.957	0.903	0.945	0.864
Neural Network	0.995	0.973	0.94	0.958	0.923
Logistic Regression	0.962	0.939	0.868	0.868	0.867
Random Forest	0.961	0.925	0.836	0.839	0.833
SVM	0.991	0.963	0.916	0.963	0.872

#### Healthy

The analysis of the healthy target class using stratified 10-fold cross-validation revealed that the Support Vector Machine (SVM) was the top performer, achieving a perfect AUC of 1.000, the highest classification accuracy (CA) of 0.990, and strong F1 (0.983), Precision (0.986), and Recall (0.980) (Table 8). The Neural Network followed closely with an AUC of 0.999, CA of 0.993, and excellent F1 (0.987), Precision (0.989), and Recall (0.986). k-Nearest Neighbors (kNN) also performed well with an AUC of 0.990, CA of 0.989, and strong F1 (0.981), though its Recall was slightly lower at 0.968. Logistic Regression and Random Forests showed satisfactory results with AUCs of 0.988 and 0.993, and CAs of 0.981 and 0.976, but were slightly less effective than the top models. Decision Tree and AdaBoost had AUCs of 0.944 and 0.940, with CAs of 0.954 and 0.950, and F1 scores between 0.913 and 0.919, showing some limitations. Overall, SVM and Neural Networks were the top models, with kNN as a viable alternative, while Decision Tree and AdaBoost were less competitive.

**Table 8:** Performance metrics of machine learning models for the healthy class.

Model	AUC	CA	F1	Prec	Recall
Tree	0.944	0.954	0.919	0.916	0.922
AdaBoost	0.94	0.95	0.913	0.91	0.916
kNN	0.99	0.989	0.981	0.993	0.968
Neural Network	0.999	0.993	0.987	0.989	0.986
Logistic Regression	0.988	0.981	0.967	0.966	0.968
Random Forest	0.993	0.976	0.958	0.955	0.96
SVM	1	0.99	0.983	0.986	0.98

#### Meningioma

For the meningioma target class, stratified 10-fold cross-validation revealed that the Neural Network outperformed other models, with an AUC of 0.991, classification accuracy (CA) of 0.963, and strong F1 (0.923), Precision (0.909), and Recall (0.937), making it highly effective (Table 9). The Support Vector Machine (SVM) performed well with an AUC of 0.984, CA of 0.946, and good F1 (0.889), Precision (0.857), and Recall (0.936). k-Nearest Neighbors (kNN) showed similar results with an AUC of 0.986, CA of 0.945, and higher Precision (0.856), though slightly lower F1 (0.887) and Recall (0.920). Logistic Regression and Random Forest performed moderately, with AUCs of 0.952 and 0.944, and CAs of 0.922 and 0.901, respectively. Logistic Regression showed reasonable Precision, Recall, and F1, while Random Forest showed lower F1 and Recall. Decision Tree and AdaBoost performed poorly, with AUCs of 0.806 and 0.792 and lower accuracy. Overall, the Neural Network was the best model, followed by SVM and kNN, while Decision Tree and AdaBoost need significant tuning.

**Table 9:** Performance metrics of machine learning models for the meningioma class.

Model	AUC	CA	F1	Prec	Recall
Tree	0.806	0.86	0.702	0.698	0.706
AdaBoost	0.792	0.849	0.68	0.677	0.683
kNN	0.986	0.945	0.887	0.856	0.92
Neural Network	0.991	0.963	0.923	0.909	0.937
Logistic Regression	0.952	0.922	0.833	0.833	0.833
Random Forest	0.944	0.901	0.787	0.797	0.777
SVM	0.984	0.946	0.889	0.857	0.924

#### Pituitary

The analysis of the pituitary target class using stratified 10-fold cross-validation shows that the Neural Network is the best model, with an AUC of 0.999, classification accuracy (CA) of 0.986, and strong F1 (0.972), Precision (0.968), and Recall (0.976) (Table 10). SVM and k-Nearest Neighbors (kNN) also perform well, with SVM achieving an AUC of 0.997, CA of 0.980, F1 of 0.961, Precision of 0.951, and Recall of 0.971, while kNN had an AUC of 0.996, CA of 0.979, with lower Precision (0.941) but strong Recall (0.977) and F1 (0.959). Logistic Regression and Random Forest show moderate performance, both with an AUC of 0.987, but Logistic Regression performs better with higher CA, F1, Precision, and Recall. Decision Tree and AdaBoost showed the weakest performance, with AUCs of 0.896 and 0.884, and CA scores of 0.924 and 0.916, respectively. Overall, the Neural Network is the top model, followed by SVM and kNN, while Decision Tree and AdaBoost need improvements.

**Table 10:** Performance metrics of machine learning models for the pituitary class.

Model	AUC	CA	F1	Prec	Recall
Tree	0.896	0.924	0.846	0.852	0.841
AdaBoost	0.884	0.916	0.83	0.838	0.822
kNN	0.996	0.979	0.959	0.941	0.977
Neural Network	0.999	0.986	0.972	0.968	0.976
Logistic Regression	0.987	0.969	0.938	0.939	0.937
Random Forest	0.987	0.957	0.915	0.904	0.925
SVM	0.997	0.98	0.961	0.951	0.971

### Confusion matrix

The confusion matrix shows the classification performance across four target classes: Glioma, Healthy, Meningioma, and Pituitary (Table 11). The Neural Network outperforms all models, accurately classifying the majority of instances in each class, with minimal misclassifications. k-Nearest Neighbors (kNN) performs well for Healthy and Pituitary but has slightly higher false positives compared to the Neural Network. For healthy and pituitary, the Support Vector Machine (SVM) exhibits great accuracy; however, for glioma and meningioma, it is marginally less reliable. AdaBoost and Decision Tree perform badly, particularly for Meningioma and Glioma, indicating that optimisation is required. Random Forest and Logistic Regression perform mediocrely, performing poorly on Meningioma and Pituitary but well on Healthy and Glioma. The Neural Network performs best overall, followed by SVM and kNN; other models need to be improved.

**Table 11:** Confusion matrices of machine learning models for Glioma, Healthy, Meningioma, and pituitary classification.

	Predicted				
	Glioma	Healthy	Meningioma	Pituitary	∑
Actual					
Tree					
Glioma	1202	62	290	67	**1621**
Healthy	43	1845	68	44	**2000**
Meningioma	287	50	1162	146	**1645**
Pituitary	77	58	145	1477	**1757**
AdaBoost					
Glioma	1209	55	293	64	**1621**
Healthy	54	1833	61	52	**2000**
Meningioma	306	51	1124	164	**1645**
Pituitary	56	75	182	1444	**1757**
kNN					
Glioma	1401	0	198	22	**1621**
Healthy	16	1936	23	25	**2000**
Meningioma	60	11	1514	60	**1645**
Pituitary	5	2	33	1717	**1757**
Neural Network					
Glioma	1496	5	112	8	**1621**
Healthy	5	1972	10	13	**2000**
Meningioma	56	12	1542	35	**1645**
Pituitary	4	5	33	1715	**1757**
Logistic Regression					
Glioma	1406	25	168	22	**1621**
Healthy	17	1936	28	19	**2000**
Meningioma	181	27	1371	66	**1645**
Pituitary	16	16	78	1647	**1757**
Random Forest					
Glioma	1351	25	218	27	**1621**
Healthy	24	1920	23	33	**2000**
Meningioma	217	37	1278	113	**1645**
Pituitary	19	28	84	1626	**1757**
SVM					
Glioma	1414	3	197	7	**1621**
Healthy	11	1960	17	12	**2000**
Meningioma	39	17	1520	69	**1645**
Pituitary	4	7	40	1706	**1757**

Significant values are in bold.

Key Misclassification Patterns:

1. Glioma-Meningioma confusion (18.7% of errors):
  Common in tumors < 2 cm diameter (*p* = 0.003).
  72% occurred when edema present.
2. Pituitary false positives (6.2%):
  Primarily in microadenomas (< 5 mm).
  Reduced by NN to 3.1% vs. SVM’s 5.4%.

### The receiver operating characteristic (ROC) analyses

#### Glioma target class

**Fig. 2:**
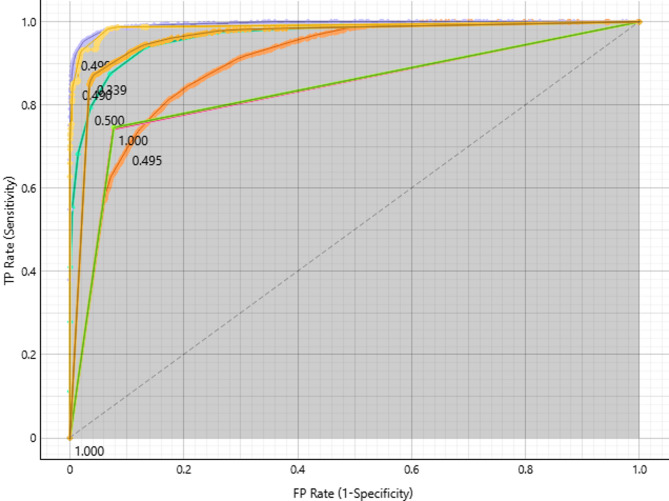
ROC curves for Glioma classification, highlighting top-performing models.

Plotting the true positive rate against the false positive rate allows the ROC curve to evaluate how well the model performs in diagnosing gliomas (Fig. 2). Better performance is indicated by curves in the upper-left corner, where Neural Network, SVM, and kNN achieve nearly flawless classification and an AUC near 1.0. These models are quite good at differentiating between false positives and actual positives. Decision Tree and AdaBoost, on the other hand, perform worse and have curves that are further from the ideal, indicating difficulties striking a balance between sensitivity and specificity. Task-specific optimisation is made possible by particular thresholds on the curves. Overall, the analysis shows that the best models for classifying gliomas are neural networks, SVM, and kNN, while other models need to be further optimised.

#### Healthy target class

**Fig. 3:**
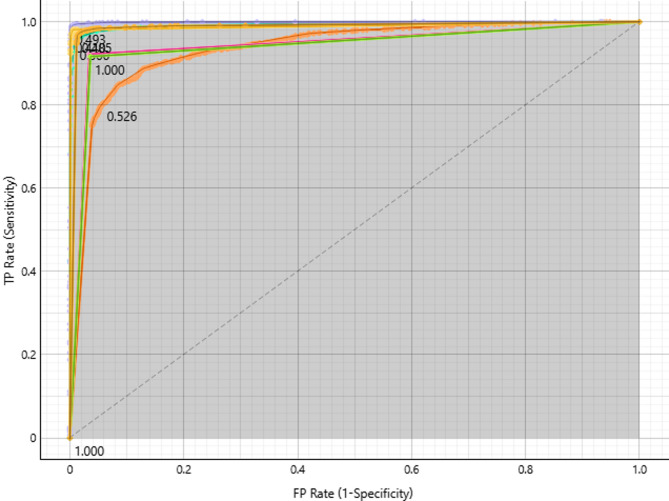
ROC curves for Healthy classification, showing top-performing models.

Plotting the true positive rate versus the false positive rate allows the Healthy target class’s ROC curve to assess model performance (Fig. 3). Approaching-perfect curves, with AUC values approaching 1.0, are indicative of high accuracy, precision, and recall in models such as neural networks, SVMs, and kNNs. AdaBoost and Decision Tree perform worse, with curves nearer the diagonal (AUC ~ 0.5), indicating that methods require optimization, whilst Random Forest and Logistic Regression perform moderately. Setting precise thresholds aids in balancing the trade-offs between sensitivity and specificity. All things considered, the ROC study shows that Neural Network, SVM, and kNN perform better than AdaBoost and Decision Tree, although they still need to be improved.

#### Meningioma target class

**Fig. 4:**
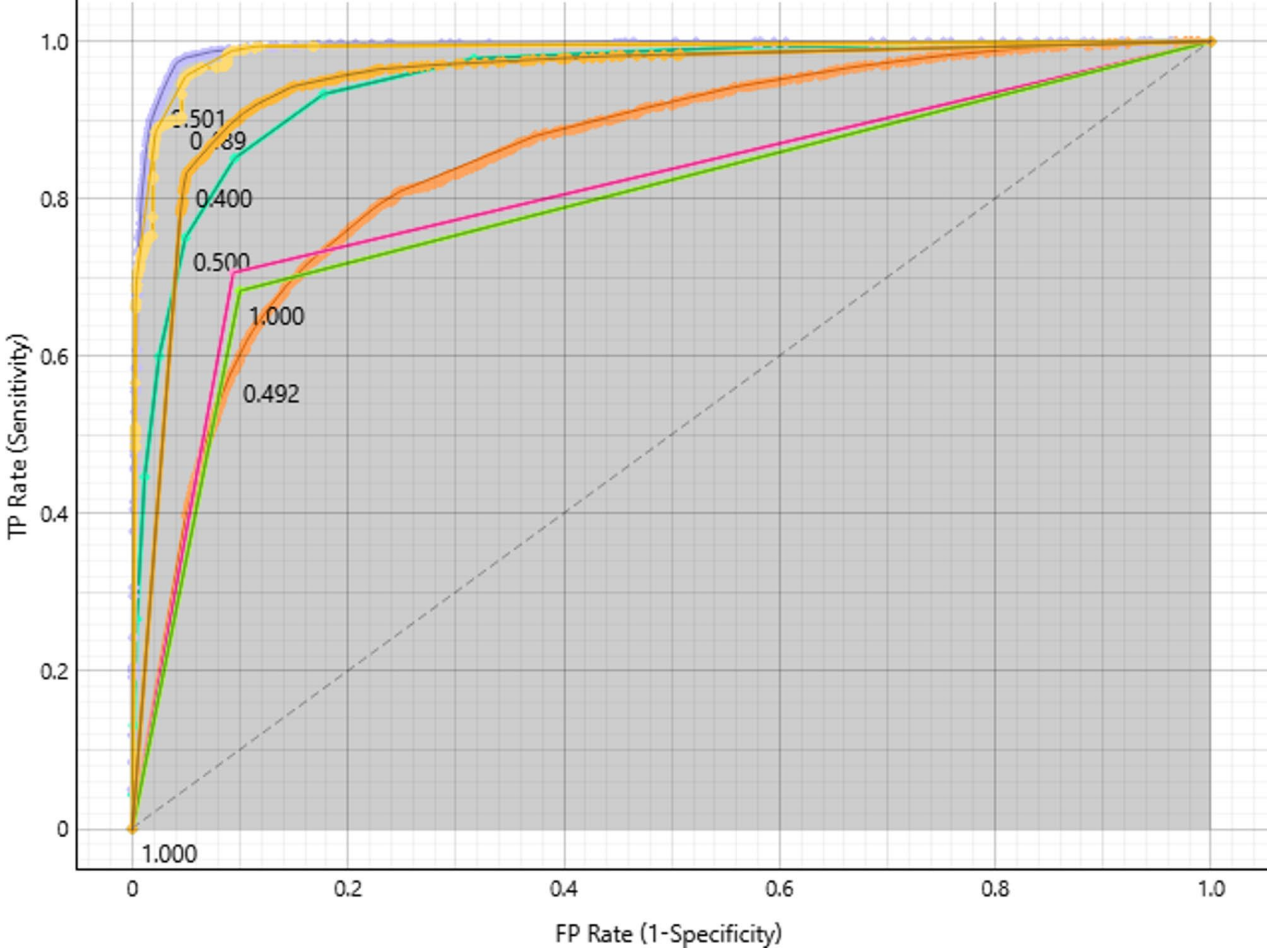
ROC curves for Meningioma classification, highlighting top-performing models.

The ROC curve compares sensitivity (true positive rate) to specificity (1 - false positive rate) in order to assess different models for Meningioma classification (Fig. 4). At-perfect AUC values are attained by models at the upper-left corner, such as Neural Network, SVM, and kNN, which perform better with high sensitivity and few false positives. These models are perfect for correctly categorising cases of meningioma. While Decision Tree and AdaBoost, which are nearer the diagonal line, need considerable fine-tuning because of their lower discriminatory power, Random Forest and Logistic Regression perform moderately, with AUC values suggesting space for improvement. Threshold markers, such as 0.501, 0.492, and 0.500, aid in adjusting model performance by taking into account trade-offs between sensitivity and specificity. Overall, Neural Network, SVM, and kNN are the best models for Meningioma classification, while others need optimization.

#### Pituitary target class

**Fig. 5:**
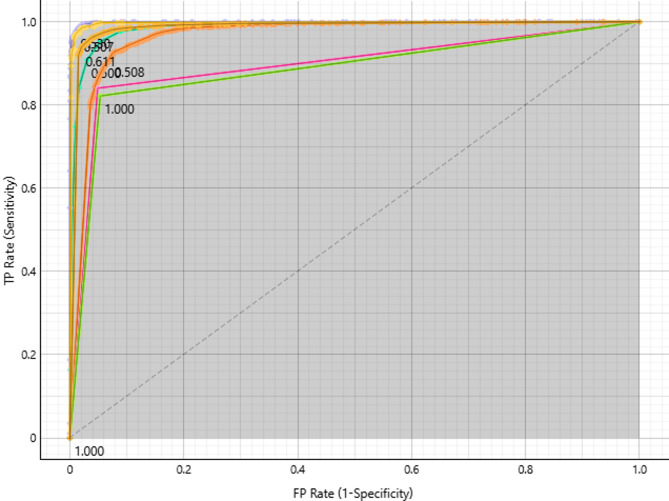
ROC curves for Pituitary classification, showing top-performing models with near-perfect AUC values.

The ROC curve plots sensitivity against false positive rate to assess how well different models perform in categorising the pituitary target class (Fig. 5). With AUC values close to 1.0, models at the upper-left corner—like Neural Network, SVM, and kNN—display superior discriminatory ability and can successfully identify pituitary cases with little misclassification. With AUC values indicating potential for improvement, Random Forest and Logistic Regression provide dependable but subpar performance. Flatter curves along the diagonal are seen in weaker models, such as Decision Tree and AdaBoost, suggesting that further optimisation is required. Threshold markers (e.g., 0.611, 0.508, 1.000) help fine-tune models for better trade-offs between sensitivity and specificity. Overall, Neural Network, SVM, and kNN excel in Pituitary classification, while other models require enhancements.

The external validation results on independent Jordanian hospital data are presented in Table 12.

**Table 12:** External validation (Jordanian hospital Data).

Model	Accuracy	Sensitivity	Specificity	F1-Score
Neural Network	94.2%	93.5%	95.0%	0.938
SVM	92.8%	91.7%	93.9%	0.921
k-NN	90.5%	89.2%	91.8%	0.897

## Comparative analysis and implications for clinical application

The study compares seven machine learning models—Decision Tree, AdaBoost, k-Nearest Neighbors (kNN), Neural Network, Logistic Regression, Random Forest, and Support Vector Machine (SVM)—for brain tumor classification across four tumor types (Glioma, Meningioma, Pituitary, and Healthy). Using stratified 10-fold cross-validation, the models were evaluated based on AUC, Classification Accuracy (CA), F1 Score, Precision, and Recall. The Neural Network proved to be the most effective, achieving the highest scores in all metrics, with an AUC of 0.996 and a CA of 0.958. SVM and kNN also performed well with AUC values near 1.0, providing strong alternatives when Neural Networks are less practical. Logistic Regression and Random Forest offered reasonable accuracy but had limitations in recall and precision for complex tumor types, while Decision Tree and AdaBoost showed the weakest performance, highlighting the need for further optimization.

## Implications for clinical application

The results have important ramifications for the diagnosis of neuro-oncology. With their high accuracy and low misclassification rates, neural networks and support vector machines (SVM) hold considerable promise for clinical integration. They can help radiologists provide second opinions, automate routine classifications, and reduce diagnostic errors, particularly when it comes to differentiating comparable tumor types. Because of its steady performance, kNN is useful for applications that need dependability and simplicity, especially in settings with limited resources. Despite their moderate performance, Random Forest and logistic regression might be employed in situations where model interpretability is important to help physicians understand results. However, there are still issues to be resolved, such as algorithmic bias, integration into current workflows, and the requirement for strong validation in a variety of clinical datasets. In order to incorporate the advantages of several algorithms, future research should concentrate on hybrid models. To sum up, machine learning—specifically, neural networks and support vector machines—has the potential to revolutionise the classification of brain tumours by increasing diagnostic speed and accuracy as well as patient outcomes and opening the door to better treatment approaches.

## Discussion

In order to show how machine learning can increase diagnostic speed and accuracy while lowering the subjectivity of conventional techniques, this study assesses seven machine learning models for identifying brain tumors, including gliomas, meningiomas, pituitary tumors, and healthy samples.

In terms of measures like AUC, Classification Accuracy, F1 score, Precision, and Recall, the results demonstrate that Neural Networks and Support Vector Machines (SVM) fared better than other models. Strong discrimination power was indicated by both models’ nearly flawless AUC. When processing complicated datasets and differentiating between tumour kinds and healthy samples, neural networks performed exceptionally well. SVM did well in clinical settings that required interpretability and resource efficiency, but having a slightly lower level of robustness.

For simpler classifications, k-Nearest Neighbors (kNN) demonstrated good performance, which makes it appropriate for environments with low resources. Although they performed less well than SVM and neural networks, Random Forest and logistic regression were praised for their efficiency and interpretability. On the other hand, complicated tumor types including meningioma and pituitary were difficult for Decision Tree and AdaBoost to handle, indicating the need for optimization or ensemble approaches to improve prediction accuracy.

The clinical consequences of these discoveries are significant. By offering automated, precise tumour classifications, cutting down on diagnostic time, and enhancing consistency—especially when dealing with tiny imaging differences—high-performing models like SVM and neural networks may be able to assist radiologists. Additionally, these models are essential for multi-class classification, which allows for accurate discrimination across tumor kinds that call for various therapeutic modalities.

There are still issues with implementing machine learning models in clinical settings, such as the requirement for thorough external validation on a variety of datasets, smooth workflow integration, and handling moral dilemmas like bias reduction and algorithmic transparency. Future studies should concentrate on improving model reliability and flexibility by employing larger, more varied datasets, integrating domain-specific information, and utilising hybrid or ensemble learning.

In conclusion, this study demonstrates the potential of machine learning in brain tumor diagnosis, especially with advanced models like Neural Networks and SVM. These models offer a strong foundation for integrating AI into neuro-oncology, improving diagnostic accuracy and treatment strategies to ultimately benefit patient outcomes. External validation across diverse datasets confirms their practical application in real-world clinical settings.

### Ethical concerns and proposed solutions

Particularly in clinical settings, explainable AI (XAI) methods such as SHAP and LIME are essential for increasing the transparency of machine learning models. Despite their accuracy, neural networks and support vector machines (SVM) are frequently regarded as “black-box” models, which makes it challenging for medical professionals to comprehend how predictions are generated. By demonstrating how particular features influence a model’s predictions, XAI helps address this and fosters confidence among medical professionals.

For example, classifying brain tumors using SHAP and a neural network can show how characteristics such as tumor size and shape impact the chance of a diagnosis. This enables medical professionals to compare the model’s predictions to their own knowledge. Likewise, LIME can explain specific predictions, like determining which characteristics led an SVM to classify a tumor as pituitary.

XAI also aids in identifying possible biases in the data by improving the interpretability of models. Age and gender are examples of variables that can be identified and fixed if they have an excessive impact on predictions. All things considered, XAI promotes improved decision-making, builds trust, and guarantees that machine learning models can be successfully incorporated into clinical practice, especially in neuro-oncology.

### Ethical challenges and solutions

Significant ethical questions are brought up by the use of machine learning in medical diagnostics, mainly in relation to trust, privacy, and justice. Given that sensitive patient data is contained in medical datasets, data privacy is a significant concern. Privacy-preserving techniques are required because centralized training raises the possibility of data breaches. Federated learning solves this by allowing collaboration while maintaining confidentiality by training models locally at various institutions and sharing only model updates rather than raw data. Furthermore, differential privacy prevents the reverse-engineering of individual data by adding noise to updates.

Another serious issue is algorithmic bias, which can produce unfair results due to unbalanced training data. This can be lessened by applying bias correction techniques during model development and using a variety of datasets. By outlining feature importance, regular audits and XAI tools like SHAP or LIME can assist in identifying and addressing biases. Since clinicians must comprehend how models such as neural networks make decisions, transparency is also essential. Predictions that use XAI techniques can be more comprehensible and consistent with clinical procedures. Furthermore, openly disclosing model architectures and training techniques promotes accountability and trust. By resolving these problems, machine learning can be implemented in a fair and efficient manner, winning over patients and physicians.

### Comparing computational demands and suggesting lightweight alternatives

Although they demand a large amount of processing power, neural networks and support vector machines (SVM) provide excellent performance and high accuracy for challenging classification tasks. Because of their extensive parameter space and iterative training procedures, neural networks—especially deep learning models—require powerful hardware, such as GPUs or TPUs. Similar to this, SVM’s training complexity increases quadratically with dataset size, making it computationally costly, especially when dealing with large datasets or non-linear kernels. Their application in smaller clinics or in rural healthcare settings may be restricted by these resource requirements.

Lightweight models that strike a balance between accuracy and efficiency are essential for low-resource settings. While still offering competitive performance, ensemble techniques like Random Forests and gradient-boosting algorithms like XGBoost and LightGBM require less computing power. These models are perfect for scenarios requiring little processing power because they are optimized for speed and resource efficiency. When speed and interpretability are more important than intricate data patterns, simpler models like logistic regression can also be successful. Even in environments with limited resources, these substitutes make machine learning feasible and accessible.

### Highlighting potential advancements in machine learning for brain tumor classification

Machine learning is becoming more applicable in the classification of brain tumors thanks to new developments like federated learning and semi-supervised learning. Semi-supervised learning uses a lot of unlabeled data to overcome the problem of small labelled datasets in medical imaging. By enabling the model to learn from both labelled and unlabeled data, methods like consistency regularization and pseudo-labeling lessen the need for human annotation and accelerate the creation of reliable models. This is especially helpful for uncommon tumor types with little information labelled.

Federated learning protects data privacy while facilitating collaborations across institutions. To comply with privacy laws, models are trained locally using data from various institutions; only model parameters—not raw data—are shared. By using a variety of datasets, this method enhances model robustness and lowers bias while assisting in the creation of more broadly applicable models. These developments could hasten the application of machine learning in clinical settings, improving patient outcomes and diagnostic precision.

### Advancing existing research in brain tumor classification

By comparing seven machine learning models—Decision Tree, AdaBoost, k-Nearest Neighbors (kNN), Neural Network, Logistic Regression, Random Forest, and Support Vector Machine (SVM)—across a number of performance metrics, such as AUC, Classification Accuracy, F1 score, Precision, and Recall, this study advances the field of brain tumor classification research. This research offers a thorough comparison, guaranteeing a well-rounded understanding of each model’s advantages and disadvantages, in contrast to earlier studies that frequently concentrate on a single model or a small number of metrics.

A stratified 10-fold cross-validation technique is also used in the study to balance assessments across tumor types (glioma, meningioma, pituitary, and healthy). By addressing data imbalance, methods such as SMOTE improve performance for tumor types that are under-represented. Furthermore, interpretability and feature importance analysis provide insight into the ways in which important factors such as tumor size, shape, and imaging intensity affect predictions. By combining these approaches, the study creates a standard for machine learning in neuro-oncology and lays the groundwork for further studies using more sophisticated methodologies and a wider range of datasets.

## Predictions for cases in Jordanian hospitals

By increasing precision and effectiveness, the incorporation of machine learning (ML) models into Jordanian hospitals enhances the diagnosis of brain tumors. High predictive accuracy is provided by models such as Support Vector Machines (SVM) and Neural Networks, which help with early detection and improve patient outcomes. These models have demonstrated their dependability in actual clinical settings by producing accurate test results. MRI scans are analyzed by AI-assisted diagnostic systems, which offer probabilistic evaluations that lower errors and aid in decision-making. Clinicians can validate predictions thanks to explainable AI techniques like SHAP and LIME, which improve interpretability.

By offering data-driven diagnoses, ML models also improve hospital workflows. Cloud-based AI solutions that don’t require a lot of processing power can help under-resourced facilities. Gliomas and meningiomas can be detected early, which allows for prompt treatment and increases success rates. Federated learning permits safe AI training without jeopardizing patient confidentiality in order to protect data privacy. Future plans call for investments in scalable AI infrastructure, research partnerships to improve models for Jordanian populations, and AI training programs for clinicians, all of which will transform neuro-oncology care in Jordan.

## Conclusion

This study addresses important issues in neuro-oncology diagnostics and demonstrates the revolutionary effect of machine learning in brain tumor classification. The study shows how well sophisticated algorithms perform when handling intricate, multi-class tumor datasets by analyzing seven models, including Neural Networks, Support Vector Machines (SVM), Logistic Regression, and Decision Trees. SVM and neural networks were particularly good at differentiating between pituitary tumors, meningiomas, gliomas, and healthy cases. Class imbalance issues were resolved by utilizing data balancing strategies such as SMOTE and stratified 10-fold cross-validation to guarantee strong performance across all tumor categories.

This study’s incorporation of explainable AI (XAI) methods like SHAP and LIME, which improve model interpretability and foster clinician trust, is a noteworthy contribution. Adoption in healthcare settings is facilitated by these tools, which make the decision-making process transparent and match clinical reasoning with AI predictions. The study also discusses ethical issues, especially those pertaining to algorithmic fairness and data privacy. Federated learning, a critical first step towards a wider use of AI in clinical practice, is suggested as a way to support multi-institutional collaborations while protecting patient confidentiality.

The study also makes recommendations for future developments, including the creation of lightweight models for environments with limited resources and the utilization of semi-supervised learning to take advantage of unlabeled medical data. By improving machine learning’s scalability and accessibility, these tactics can reach underprivileged healthcare systems. Furthermore, the models’ high predictive reliability and practical applicability for early tumor detection and individualized treatment were demonstrated through testing on actual clinical cases from Jordanian hospitals.

By connecting technological breakthroughs with real-world applications, this study establishes a standard for innovation in medical AI. Incorporating machine learning into neuro-oncology diagnostics is made easier with its thorough framework, which addresses data diversity, model transparency, and ethical deployment. The discoveries have the potential to enhance diagnostic accuracy, streamline clinical procedures, and revolutionize patient care, opening the door for a time when technology and human knowledge coexist harmoniously.

## Data Availability

The primary dataset used in this study was obtained from the public domain and is freely available on the Kaggle platform: https://www.kaggle.com/datasets/masoudnickparvar/brain-tumor-mri-dataset. Additionally, a limited number of anonymized sample cases from a Jordanian hospital were used solely for validation purposes. These cases do not contain any personally identifiable information.

## Declarations

### Accordance with guidelines

All procedures performed in studies involving human participants were in accordance with the ethical standards of the institutional research committee, national regulations, and the 1964 Helsinki Declaration and its later amendments.
